# Isolation and Quantification of Uremic Toxin Precursor-Generating Gut Bacteria in Chronic Kidney Disease Patients

**DOI:** 10.3390/ijms21061986

**Published:** 2020-03-14

**Authors:** Tessa Gryp, Geert R.B. Huys, Marie Joossens, Wim Van Biesen, Griet Glorieux, Mario Vaneechoutte

**Affiliations:** 1 Department of Internal Medicine and Pediatrics, Nephrology Section, Ghent University Hospital, 9000 Ghent, Belgium; wim.vanbiesen@ugent.be (W.V.B.); griet.glorieux@ugent.be (G.G.); 2 Department of Diagnostic Sciences, Laboratory Bacteriology Research, Ghent University, 9000 Ghent, Belgium; mario.vaneechoutte@ugent.be; 3 Department of Microbiology, Immunology and Transplantation, Molecular Microbiology—Microbiome Research Lab, KU Leuven, 3000 Leuven, Belgiummarie.joossens@kuleuven.vib.be (M.J.)

**Keywords:** qPCR, bacterial culture, fecal bacteria, chronic kidney disease, protein-bound uremic toxins, bacterial metabolization, aromatic amino acids, *p*-cresol, indole, indole-3-acetic acid, phenol

## Abstract

In chronic kidney disease (CKD), impaired kidney function results in accumulation of uremic toxins, which exert deleterious biological effects and contribute to inflammation and cardiovascular morbidity and mortality. Protein-bound uremic toxins (PBUTs), such as *p*-cresyl sulfate, indoxyl sulfate and indole-3-acetic acid, originate from phenolic and indolic compounds, which are end products of gut bacterial metabolization of aromatic amino acids (AAA). This study investigates gut microbial composition at different CKD stages by isolating, identifying and quantifying PBUT precursor-generating bacteria. Fecal DNA extracts from 14 controls and 138 CKD patients were used to quantify total bacterial number and 11 bacterial taxa with qPCR. Moreover, isolated bacteria from CKD 1 and CKD 5 fecal samples were cultured in broth medium supplemented with AAA under aerobic and anaerobic conditions, and classified as PBUT precursor-generators based on their generation capacity of phenolic and indolic compounds, measured with U(H)PLC. In total, 148 different fecal bacterial species were isolated, of which 92 were PBUT precursor-generators. These bacterial species can be a potential target for reducing PBUT plasma levels in CKD. qPCR indicated lower abundance of short chain fatty acid-generating bacteria, *Bifidobacterium* spp. and *Streptococcus* spp., and higher *Enterobacteriaceae* and *E. coli* with impaired kidney function, confirming an altered gut microbial composition in CKD.

## 1. Introduction

In chronic kidney disease (CKD), impaired kidney function results in the accumulation of uremic toxins [1,2,3], which exert deleterious biological effects and contribute to inflammation and cardiovascular morbidity and mortality [4,5,6,7,8]. Well-known protein-bound uremic toxins (PBUTs), *p*-cresyl sulfate (*p*CS), *p*-cresyl glucuronide (*p*CG), indoxyl sulfate (IxS) and indole-3-acetic acid (IAA) originate in the colon, where aromatic amino acids (AAA) are metabolized by gut bacteria into phenolic (*p*-cresol and phenol) and indolic compounds (indole and IAA) [9,10,11,12]. *p*-Cresol and indole are sulfated in the colon mucosa and by the liver into *p*CS and IS, and a small part is glucuronidated into *p*CG [9,13,14], while IAA enters the circulation without further modifications [15,16,17]. These PBUTs reversibly bind to plasma albumin [18]; however, only the free fraction can be removed by dialysis [19,20].

During recent years, there is increasing evidence that the gut bacterial composition is altered in patients with CKD [21,22,23,24,25,26,27,28,29,30]. Generally, in CKD, increased growth of both aerobes and anaerobes takes place in the small intestine [24,25], while in the colon the numbers of aerobic bacteria (e.g.*, Enterobacteriaceae*) are increased [23,30] and anaerobic bacteria (e.g.*,* bifidobacteria) are decreased [23,29,30]. The increased aerobic bacterial growth is a consequence of an elevated pH resulting from the high ammonia levels in the gut in CKD [31]. Ammonia is a bacteria-derived hydrolysis end product of urea, which accumulates in the circulation and enters the gut lumen via the entero-hepatic cycle [31] and by diffusion through the disrupted intestinal epithelial barrier as is typically seen in patients with CKD [32,33]. This leads to a uremic milieu in the gut lumen, altering the gut microbial composition in favor of urease expressing bacteria in end stage kidney disease (ESKD) patients [27]. However, in a recent study by our group [34], the generation of fecal PBUT precursor metabolites by fecal bacteria did not alter with CKD progression nor compared to controls. This suggests that the accumulation of PBUTs in plasma is mainly due to the impaired kidney function itself.

Nevertheless, minimizing the generation by gut bacteria of phenolic and indolic compounds in the hope to subsequently reduce plasma PBUT levels remains an important goal due to the absence of other adequate therapies to reduce the plasma levels of PBUTs in patients with CKD. It is known that certain bacterial isolates are capable to generate PBUT precursors in an in vitro setting [9,35,36,37,38,39,40], but the generation capacity of fecal bacteria isolated from patients with CKD has not yet been investigated. Numerous studies in patients with CKD already addressed the idea of applying pro-, pre- and synbiotics [30,41,42,43,44,45,46] to increase saccharolytic and decrease proteolytic bacteria. Thus far, only one specific PBUT has been targeted [47]. In addition, it has been shown that dietary intake is the most important determinant of the fecal metabolome in CKD, next to the loss of kidney function [13], suggesting that diet could also play an important role in altering the gut bacterial composition in a favorable way. Intake of low-protein diets lowers the serum levels of IxS and the generation of various phenolic and indolic metabolites in healthy humans and mice [48].

In this study, bacterial isolates obtained from fecal samples from patients with CKD stage 1 and stage 5 were taxonomically characterized and metabolically classified as PBUT precursor-generating bacteria based on their capacity to assimilate AAA into phenolic (*p*-cresol and phenol) and/or indolic (indole and IAA) compounds. In addition, the abundance of specific fecal PBUT precursor-generating bacteria in the different stages of CKD and in controls was investigated.

## 2. Results

### 2.1. Culture Media to Isolate Protein-Bound Uremic Toxin Precursor-Generating Bacteria from Fecal Samples

To isolate PBUT precursor-generating bacteria, an appropriate culture medium had to be selected. Therefore, seven different media were compared based on the cultivation of a bacterial test panel consisting of 12 known uremic toxin precursor-generating bacterial species (Figure S1), and the cultivation of fecal bacteria from whole fecal samples (Table S1). Besides the commercially available Schaedler medium (SCH, Becton Dickinson, Erembodegem, Belgium), the yeast casitone fatty acid glucose (YCFAG) medium [37,49] was used as the basis for another six media, of which specific components were omitted separately, to check their importance for the cultivation of UT precursor-generating bacteria. Moreover, the effect of the supplementation of AAA and of a fecal suspension from healthy volunteers (FS) was investigated.

All bacterial species from the test panel were culturable with the 24 YCFAG media combinations, except for *Bifidobacterium* sp. and *Lactobacillus* sp., which only grew when AAA and FS were added to the complete YCFAG (YCFAG Δ0), YCFAG without vitamins (YCFAG ΔV) or YCFAG without short chain fatty acids (YCFAG ΔS). For the SCH medium, addition of AAA was detrimental for the growth of most tested bacterial species, except *B. longum*, *E. coli*, *L. paracasei*, and *S. epidermidis*. SCH medium, with and without the supplementation of FS, resulted in the growth of all species of the test panel (Figure S1). Based on these results, the five medium formulations that supported growth of all bacterial species (i.e.*,* YCFAG Δ0 +AAA +FS, YCFAG ΔV +AAA +FS, YCFAG ΔS +AAA +FS, SCH and SCH + FS) were used further to culture bacteria from fecal samples. Table S1 provides an overview of all microorganisms that could be cultured on these five media. Most anaerobic and aerobic bacteria were isolated from YCFAG Δ0 +AAA +FS and from SCH, and these media were further used to isolate PBUT precursor-generating bacteria from fecal samples of patients with CKD.

### 2.2. Isolation and Identification of Protein-Bound Uremic Toxin-Generating Bacteria from Fecal CKD Samples

Figure S2 illustrates an overview of the isolation of PBUT precursor-generating bacteria. In total, 148 different bacterial species were isolated from CKD 1 (*n* = 6) and CKD 5 (*n* = 6) fecal samples to cover fecal bacteria representative for both early and late stage of CKD. Of these, 99 species could be identified by means of MALDI-TOF (Bruker, Mannheim, Germany) and 92 were considered as PBUT precursor-generating bacteria when, for at least one of the precursor metabolites (i.e.*, p*-cresol, phenol, indole or IAA), the levels were above the limit of detection (LOD) as assessed by U(H)PLC. Table 1 lists all isolated bacterial species with their generation capacity of PBUT precursors under aerobic and anaerobic conditions. Generally observed, *p*-cresol and phenol were mainly generated under anaerobic conditions, while indole and IAA could be generated under both aerobic and anaerobic conditions. Phenolic compounds and IAA were predominantly generated by bacterial species belonging to the *Actinomycetaceae*, *Bacteroidaceae*, *Clostridiaceae, Enterococcaceae, Lachnospiraceae, Staphylococcaceae* and *Tannerellaceae*, while indolic compounds were mainly generated by the *Bifidobacteriaceae, Coriobacteriaceae*, *Enterobacteriaceae*, *Propionibacteriaceae* and *Rikenellaceae*. In addition, *Bacteroides uniformis*, *Odoribacter splanchnicus* and *Oscillibacter* sp. are the only bacterial species capable of generating all four PBUT precursor metabolites under anaerobic conditions.

### 2.3. Quantification of Fecal Bacterial Species in Different Stages Of CKD

**Figure 1:**
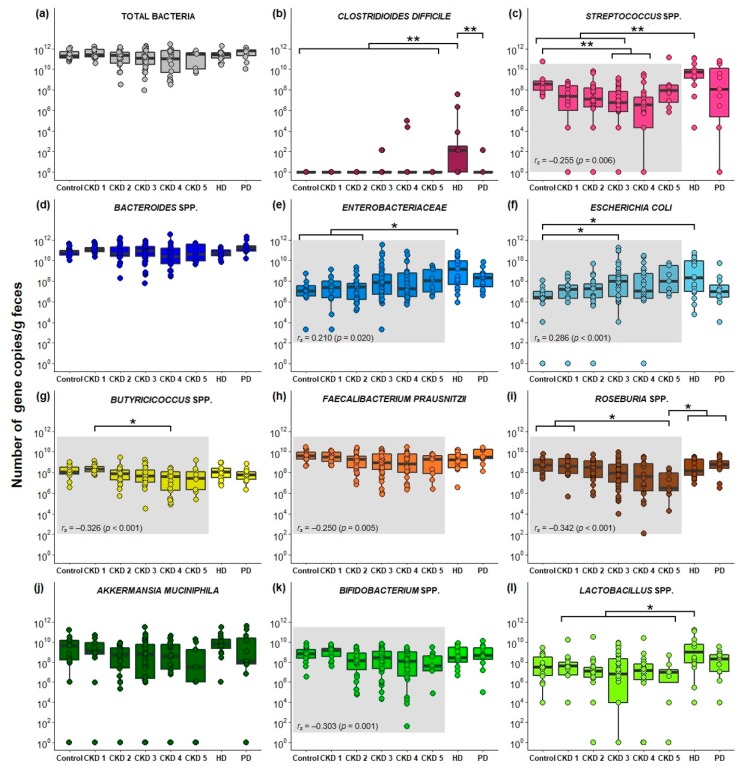
Bacterial gene copies per gram feces per chronic kidney disease (CKD) stage: (**a**) Total bacterial 16S rRNA; (**b**) *Clostridioides difficile*; (**c**) *Streptococcus* spp.; (**d**) *Bacteroides* spp.; (**e**) *Enterobacteriaceae*; (**f***) Escherichia coli*; (**g**) *Butyricicoccus* spp.; (**h**) *Faecalibacterium prausnitzii*; (**i**) *Roseburia* spp.; (**j**) *Akkermansia muciniphila*; (**k**) *Bifidobacterium* spp.; (**l**) *Lactobacillus* spp. *: *p* ≤ 0.05; **: *p* ≤ 0.001; *r_s_*: Spearman’s rank test; gray square: correlation between respective bacteria versus CKD stages 1–5 and control.

Quantitative PCR (qPCR) analysis was assessed in different stages of CKD and in controls for 11 bacterial taxa, and the total number of 16S rRNA gene copies with a universal bacterial qPCR (Figure 1). Prior to statistical analysis, one CKD 1 sample was excluded due to the absence of qPCR signal for each of the performed assays (LOD > Concentration). The abundance of *Bacteroides* spp., *Bifidobacterium* spp., *Faecalibacterium prausnitzii* and *Akkermansia muciniphila*, as well as the total number of bacterial 16S rRNA gene copies was comparable among the different stages of CKD and the control group. In fecal samples from hemodialysis (HD) patients, a higher abundance of *Streptococcus* spp. and *Enterobacteriaceae* was found compared to controls and early stages of CKD, and for *Escherichia coli* compared to controls. Moreover, compared to HD patients, the abundance of *Lactobacillus* spp. decreased in CKD stage 1–5 patients, and the abundance of *Clostridioides difficile* in all other CKD stages and controls. Decreased levels of *Roseburia* spp. were observed in CKD 5 compared to controls, CKD 1, HD and peritoneal dialysis (PD) patients. In addition, a lower abundance of *Streptococcus* spp. was found in CKD 3 and CKD 4 compared to controls, while the opposite was found for *E. coli*, i.e., an increase in CKD 3 compared to control. Finally, decrease in kidney function (ranging from control to CKD 5) positively correlated with the abundance of *Enterobacteriaceae* (*r_s_* = 0.210), and *E. coli* (*r_s_* = 0.286), while an inverse correlation was found with *Streptococcus* spp. (*r_s_* = −0.255), *Butyricicoccus* spp. (*r_s_* = −0.326), *F. prausnitzii* (*r_s_* = −0.250), *Roseburia* spp. (*r_s_* = −0.342) and *Bifidobacterium* spp. (*r_s_* = −0.303) (Figure 1; all *p* < 0.05). Similar results were obtained when correlating the abundance of the bacterial taxa with estimated glomerular filtration rate (eGFR), except for *Streptococcus* spp. (Table S2).

**Figure 2:**
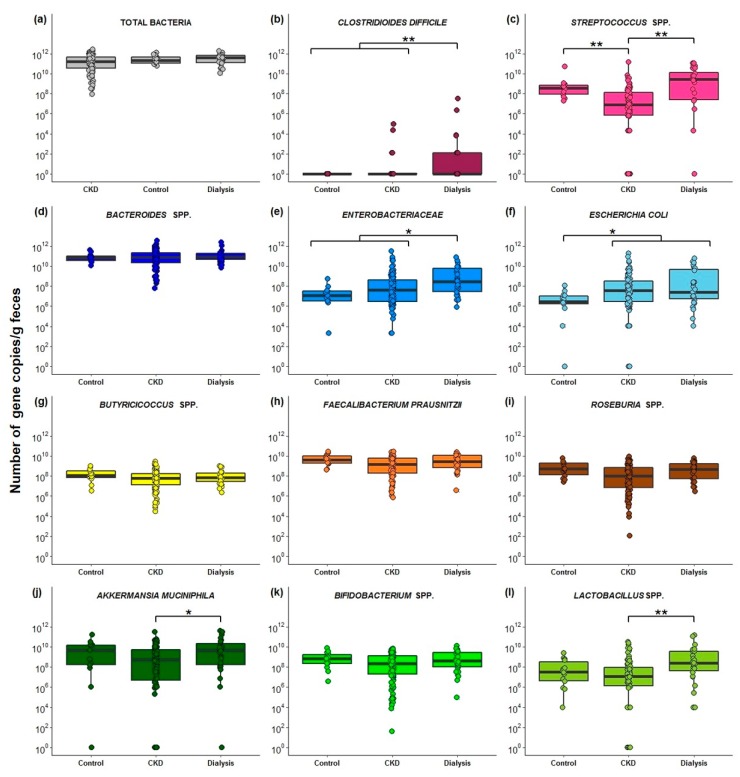
Bacterial gene copies per gram feces for the control, CKD and dialysis group: (**a**) Total bacterial 16S rRNA; (**b**) *Clostridioides difficile*; (**c**) *Streptococcus* spp.; (**d**) *Bacteroides* spp.; (**e**) *Enterobacteriaceae*; (**f***) Escherichia coli*; (**g**) *Butyricicoccus* spp.; (**h**) *Faecalibacterium prausnitzii*; (**i**) *Roseburia* spp.; (**j**) *Akkermansia muciniphila*; (**k**) *Bifidobacterium* spp.; (**l**) *Lactobacillus* spp. *: *p* ≤ 0.05; ** *p* ≤ 0.001.

Comparison of the total bacterial number and the abundance of the 11 PBUT precursor-generating bacterial taxa in the control, CKD (CKD stages 1 to 5) and dialysis groups (HD and PD patients), revealed no significant differences for the total bacterial 16S rRNA gene copies, the abundance of *Bacteroides* spp., *Bifidobacterium* spp., *Butyricicoccus* spp., *F. prausnitzii*, and *Roseburia* spp. (Figure 2). However, a higher abundance was found in the dialysis group compared to CKD for *A. muciniphila*, *C. difficile*, *Enterobacteriaceae*, *Lactobacillus* spp., and *Streptococcus* spp., and compared to controls for *C. difficile*, *Enterobacteriaceae*, and *E. coli*. In addition, in CKD, the abundance of *Streptocococcus* spp. decreased and of *E. coli* increased compared to the control group (Figure 2).

## 3. Discussion

The gut microbiota is responsible for the generation of PBUTs [10,12,51], which accumulate in the blood circulation of patients with CKD [1,2,3]. At the moment, no effective therapies exist to reduce the plasma levels of these PBUTs. This is the first study to isolate and identify PBUT precursor-generating bacteria from fecal samples from patients with CKD stage 1 and 5 patients. These results give us more insight regarding which bacteria are responsible for the metabolization of aromatic amino acids into phenolic (i.e.*, p*-cresol and phenol) and indolic (i.e.*,* indole and IAA) compounds. In addition, 11 PBUT precursor-generating bacterial taxa and the total number of bacteria were quantified, using qPCR, in patients with different stages of CKD and healthy controls.

In total, 148 different bacterial species were isolated, of which 92 could be classified as PBUT precursor-generating bacteria. Moreover, to our knowledge, 51 of the isolated bacterial species were not previously described as generators of phenolic or indolic compounds. Fecal proteolytic bacterial species predominantly belong to the genera *Bacteroides* and *Propionibacterium*, and although with fewer representatives, also to the genera *Bacillus*, *Clostridium*, *Staphylococcus* and *Streptococcus* [52]. This is in line with the findings of the present study, in which the *Bacteroidaceae*, *Clostridiaceae, Lachnospiraceae* and *Staphylococcaceae* were the predominant generators of phenolic compounds in addition to IAA. In the current study, these metabolites were also generated by bacterial species belonging to the *Actinomycetaceae*, *Tannerellacae*, and *Enterococcaceae,* of which the latter family also was classified as a phenolic compound-generating bacterial taxon [9]. Most fecal species with the capacity to generate phenolic compounds belong to the genus *Bacteroides*, although species-to-species differences have been reported [37,40,53]. These differences in the capacity to generate phenolic compounds by *Bacteroides* spp. can be explained by the different compositions of the culture media used and thus by a different substrate availability, which determines the metabolization pathway of gut bacteria (carbohydrate or amino acid metabolization). This has already been reported for *Bifidobacterium* spp., which can switch to the metabolization of amino acids in the absence of carbohydrates [36]. Because of the presence of the tyrosine deaminase gene, *Clostridiaceae* and *Enterobacteriaceae* also have the property of converting tyrosine into *p*-cresol, which were increased in ESKD patients [27]. However, in the present study, the generation of *p*-cresol was only observed in bacterial species belonging to the *Clostridiaceae*. Moreover, in the present study, bacterial species generating solely IAA belong to the *Bacillaceae*, *Bifidobacteriaceae*, *Brevibacteriaceae*, *Coriobacteriaceae, Corynebacteriaceae, Eggerthellaceae, Microbacteriaceae* and *Micrococcaceae*, while the other bacterial species mostly also produce indole and/or phenolic compounds. Conversion of tryptophan into indole is performed by tryptophanase, an enzyme known to be expressed by (at least) 27 gut-associated genera [54]. Tryptophanase activity is also present in *Clostridiaceae*, *Enterobacteriaceae* and *Verrucomicrobia*, which all have been reported to be elevated in ESKD patients [27]. Interestingly, in this study, most of the *Lactobacillus* spp. generate none of the measured indolic and phenolic compounds, but are potent generators of lactic acid and phenyl lactic acid [55,56]. In healthy conditions, most abundant *Lactobacillus* spp. in the colon include *L. casei*, *L. delbrueckii*, *L. plantarum*, *L. rhamnosus*, and *L. ruminus* [57], with the exception of *L. plantarum*, all of these were also isolated from the CKD fecal samples in this study.

Based on the bacterial generation of phenolic and indolic compounds in vitro and the associations between bacterial taxa with high *p*CS or high IxS serum levels in HD patients [58], 11 bacterial taxa were selected to explore the abundance of these PBUT precursor-generators in different stages of CKD. Altered gut microbial composition in CKD was observed by Vaziri et al., revealing a qualitative difference of 190 operational taxonomic units between HD patients and controls [21]. A change in the gut microbial composition is confirmed in our CKD cohort with changes in the abundance of *Butyricicoccus* spp., *C. difficile*, *Enterobacteriaceae*, *E. coli*, *Lactobacillus* spp., *Roseburia* spp. and *Streptococcus* spp. in the different stages of CKD and control. Moreover, a decline in kidney function was positively associated with *Enterobacteriaceae* and *E. coli*, and negatively associated with *Bifidobacterium* spp., *Butyricicoccus* spp., *F. prausnitzii*, *Roseburia* spp. and *Streptococcus* spp.. However, the total number of bacterial 16S rRNA gene copies was similar among all CKD stages and controls, in line with two other studies comparing (non-)dialyzed ESKD patients with controls [29,59]. In a cultured-based approach to estimate the total bacterial number, no significant differences were observed between HD and controls, either [30], while in another culture study, a lower total number of bacteria were observed in CKD and HD patients [23]. For *Bacteroides* spp., we found comparable amounts over the different CKD stages and controls, which was also reported for *B. fragilis* in PD patients compared to controls [28]. *Bacteroides* is one of the most abundant bacterial genera in the gut of healthy adults and patients with CKD [60]. Enrichment of aerobic bacteria in the colon of patients with CKD has been frequently reported [23,29,30,61,62], and was also confirmed in the present study (i.e.*, Enterobacteriaceae* and *E. coli*). However, this was not the case for all studies assessing the gut microbial composition in CKD. A qPCR-based study found no differences in *E. coli* levels in PD patients and controls [63], while another study found decreased *E. coli* levels in Chinese ESKD patients [29]. In addition, in this study, the abundance of *Streptococcus* spp. decreased with impaired kidney function, and increased again under dialysis therapy. Similar results were found in the study of Stadlbauer et al., with higher *Streptococcus* spp. levels in HD [62] and type 2 diabetes mellitus CKD patients [64] compared to controls. Elevated levels of *Streptococcus* spp. have been associated with a range of human infections [65].

The previously observed decrease of anaerobic bacteria in CKD [23,29] was also confirmed in the present study with the decrease of *Bifidobacterium* spp. and the inverse correlation of *Bifidobacterium* spp., *Butyricicoccus* spp., *F. prausnitzii* and *Roseburia* spp. with impaired kidney function. Bifidobacteria and lactobacilli are commonly used probiotics, aiming at health-promoting effects to the host and commensal gut microbiol homeostasis [66]. In our CKD cohort, the abundance of *Lactobacillus* spp. was higher in HD patients compared to patients with CKD stages 1 to 5. In a previous sequence-based comparison of the gut microbiota between patients with CKD stages 1 to 5 and non-CKD controls; however, higher *Lactobacillus* spp. levels in CKD were found [60]. Another health-promoting bacterial species is *Akkermansia muciniphila*, an essential propionate-generating and mucus-degrading species that is beneficial through the production of energy from mucus degradation and as such supporting the growth of butyrate-producing bacteria [60,67,68,69]. A sequencing study comparing CKD patients with controls reported a strong negative correlation with CKD and a reduction of the genus *Akkermansia*, of which the species *A. muciniphila* is the only human representative [60]. These findings are in contrast to the present study with only a lower abundance of *A. muciniphila* in the CKD group versus the dialysis group, and no inverse correlation with loss of kidney function. Other short chain fatty acid (SCFA)-generating bacteria include *Butyricicoccus* spp., *Faecalibacterium prausnitzii* and *Roseburia* spp., which predominantly colonize the colonic mucus layer and particularly generate butyrate. Butyrate is beneficial for the health because it is an energy source for colonocytes and it promotes cell differentiation, suppresses colonic inflammatory response, decreases luminal pH and improves tight junction assembly [59,69,70,71]. Our results show that the three aforementioned butyrate-producing taxa were inversely associated with impaired kidney function. This is consistent with previous findings observing a decrease of butyrate-generating bacteria in ESKD patients compared to controls [29,61,62,72] and depletion of microbial families possessing butyrate-forming enzymes in ESKD patients [27]. However, in the study of Terpstra et al., the abundance of *F. prausnitzii* and *Roseburia* spp. was comparable in ESKD patients and controls [59]. Finally, the pathogenic bacterium *C. difficile*, which is related to severe antibiotic-associated diarrhea [73,74] and a well-known producer of *p*-cresol [39,40], was elevated in the HD group of the present study. This increased abundance could be explained by elevated antibiotic intake due to a higher infection rate in ESKD patients [74].

*p*-Cresol and indole originate from two independent metabolization pathways [9,17], which are associated with a completely different gut microbial composition [58]. *p*-Cresol exerts various toxic effects to biological functions [75,76,77,78,79,80,81,82]. This is in contrast to indole, which acts as an intercellular signal molecule and contributes to the immune cell homeostasis and the host-microbe homeostasis at the mucosal surface by promoting gene expression of gut epithelial cell junctions [17,35,83]. Of interest, only a small fraction of tryptophan (~5%) in the gut is converted into indole, while the other fraction is used in the kynurenine (~95%) and serotonin (~1-2%) pathway [17,54]. The beneficial properties of indole imply that it is less desirable to reduce the generation of indole compared to the generation of *p*-cresol, and in this context, the differentiated metabolization pathways of *p*-cresol and indole could be considered as an advantage rather than a disadvantage. Pro-, pre- and synbiotics could play an important role in minimizing the generation of only *p*-cresol and thus also of the plasma levels of *p*CS, which is supported by numerous intervention studies in patients with CKD, showing only a reduction of plasma *p*CS or IxS levels, but not of both toxins [30,41,42,43,44,45,46]. Of note, a decrease of aerobic bacteria, generating mostly indolic compounds, could be beneficial to the gut microbial homeostasis considering the present aerobic overgrowth in CKD. These elevated levels of aerobes, shown to inhibit anaerobic bacterial growth [23,30], could also negatively interfere with the activity of SCFA-generating bacteria.

As shown in the present study, phenolic compounds were mostly generated by bacterial species from the phyla Bacteroidetes (i.e.*, Actinomycetaceae*, *Bacteroidaceae*, *Odoribacter splanchnicus*, *Prevotella copri* and *Tannerellaceae*) and Firmicutes (i.e.*, Clostridiaceae*, *Enterococcaceae*, *Lachnospiraceae*, *Oscillibacter* sp., *Staphylococcaceae* and *Streptococcaceae*). Quantification of *Bacteroides* spp., *C. difficile* and *Streptococcus* spp. with qPCR revealed elevated levels of *C. difficile* and *Streptococcus* spp. in HD patients which marks both taxa as potential targets to reduce *p*-cresol generation in patients with CKD. *Bacteroides* spp. did not change in abundance in CKD versus controls, but could also be a target as these have the capacity to generate phenolic compounds. To our knowledge, no interventions have so far been assessed to reduce one of these bacterial taxa in patients with CKD or in healthy controls. Because our results indicate that both *Bifidobacterium* spp. and *Lactobacillus* spp. are reduced with impaired kidney function and although they are known to generate only IAA, these taxa may be potential probiotics. In CKD, numerous studies have already addressed the impact of these bacterial species on the reduction of plasma *p*CS and IxS levels and their gut-derived precursor metabolites [30,41,42,43,44,45,46,47,84,85]. Fecal *p*-cresol, fecal indole and plasma IxS could be reduced by a combination of antibiotic-resistant lactic acid-generating bacteria (i.e.*, B. infantis*, *L. acidophilus* and *Enterococcus faecalis*) [30]. In addition, a synbiotic treatment, consisting of *B. breve* Yakult, *L. casei* Shirota and oligofructose-enriched inulin (OF-IN), reduced urinary *p*-cresol, a proxy for colonic *p*-cresol generation, and promoted the growth of bifidobacteria in healthy volunteers [84]. This is in line with the SYNERGY trial, showing elevated levels of bifidobacteria in CKD stage 4 and stage 5 patients after intake of a synbiotic containing galactooligosaccharides (GOS), OF, *Bifidobacterium* spp., *Lactobacillus* spp. and *Streptococcus* spp. [41]. Moreover, in the same study, excluding the patients receiving antibiotic treatments, the relative abundance levels of *F. prausnitzii* increased and serum *p*CS levels decreased in the patients with CKD [41]. Another study assessed the stimulation of saccharolytic fermentation and the reduction of proteolytic fermentation attributed by the combination of *L. casei* Shirota and OF-IN [47]. Elevation of SCFA-generating bacteria through high total dietary fiber intake may contribute to the reduction of microinflammation present in CKD, and could hamper the progression of CKD [72,86]. In HD patients, plasma *p*CS levels could be reduced by consumption of the prebiotic OF-IN [42] and intake of the synbiotic containing *L. casei* Shirota, *B. breve*, GOS, lactose and monosaccharide [44]. Moreover, the synbiotic Probinul-neutro^®^ (CadiGroup, Rome, Italy) reduced plasma *p*CS levels in CKD stage 3 and stage 4 [45], while levels of IxS could be reduced by resistant starch [46] and by *B. longum* [43] in HD patients. These studies indicate promising therapeutic interventions to reduce fecal *p*-cresol and plasma *p*CS and IxS levels in CKD. Moreover, even in earlier CKD stages, already associated with a change in the gut microbial composition [87], which might delay the progression of the disease. In a recent paper by our group [34], regarding the same study population, the protein intake was estimated by calculating the ratio of urinary urea over urinary creatinine, a proxy for protein intake. This ratio was not significantly different among the different stages of CKD nor in comparison to the control group and therefore we cannot attribute the observed changes in gut bacterial composition to differences in protein intake in this study population.

A possible shortcoming of this study was the assessment of the gut microbial community by only qPCR, depending on a selection of gut bacterial taxa, and not of the entire microbial community by means of high-throughput molecular techniques. However, our qPCR assays indicate an alteration in the gut microbial composition over the different stages of CKD and controls for nine bacterial taxa, of which some are at species level. However, in a previous microbiome study by our research group, the gut microbial composition of the HD patients from the current CKD cohort was analyzed with phylogenetic community profiling and revealed a similar microbial profile, based on genera and higher taxonomic taxa, was observed with that of controls [58]. This was in line with another sequencing study comparing ESKD patients with controls [29], but not in a study comparing controls with non-dialyzed and dialyzed CKD patients [61,88]. The sequencing results of the CKD group are in preparation for another paper, in view of the extent of the data.

In summary, isolation and quantification of PBUT precursor-generating bacteria from fecal samples of patients with CKD (stages 1 and 5) showed that phenolic compounds are mostly generated by anaerobic bacteria whereas indolic compounds are produced by both anaerobes and aerobes. The present study is one of the first investigating PBUT precursor-generating bacterial concentrations with qPCR in all stages of CKD, and indicated that kidney function decline is accompanied by a decrease in the abundance of SCFA-generating bacteria, *Bifidobacterium* spp. and *Streptococcus* spp., and an increase of aerobic bacteria, i.e., *Enterobacteriaceae* and *E. coli*. Our findings might trigger the development of potential interventions to minimize the generation of especially *p*-cresol by the gut microbiota and subsequently to reduce plasma *p*CS levels in patients with CKD. Moreover, in the future, these bacterial species with the capacity to generate PBUT precursor metabolites need to be assessed in in vitro models and in vivo studies to elucidate their pathophysiological role, e.g., their effect on the intestinal barrier function and inflammation, in CKD.

## 4. Materials and Methods

### 4.1. Study Population and Sample Collection

The study population, from which a fecal sample was collected, has been previously used and described, including the clinical parameters, by the authors [34]. Briefly, in total, 14 healthy controls, 111 non-dialyzed CKD patients (stages 1–5), 16 HD patients, and 11 PD patients were included. Exclusion criteria were active infection (C-reactive protein, CRP > 20mg/L), immunosuppressive therapy, obesity (Body Mass Index > 35), inflammatory bowel disease, active malignancy, cardiovascular event in the past three months, pregnancy, transplantation, use of non-steroidal anti-inflammatory drugs within the past month, vascular access problems for HD patients, and age < 18 years. Before inclusion, all patients and healthy controls gave written informed consent. The study was conducted in accordance with the Declaration of Helsinki, and approved by the Medial Ethics Committee of the Ghent University Hospital (Ref 2010/033, B67020107926 and Ref 2012/603, B670201214999).

### 4.2. Culture Media

Prior to the isolation of PBUT precursor-generating bacteria, appropriate culture media were selected. For this purpose, a total of 28 different culture broths (Figure S1) were compared with respect to their ability to support growth of a test panel, composed of bacterial species known to generate *p*-cresol and/or indole. AAA (total concentration of 2.5 mM l-tyrosine, 2.5 mM l-phenylalanine and 2.5 mM l-tryptophan, Sigma Aldrich, Saint Louis, MO, USA) were added to stimulate the growth of proteolytic (fermentation) bacteria. Moreover, to mimic the natural gut environment of the fecal bacteria, a sterile FS was prepared and added to some of the media. For this, whole fresh fecal samples (*n* = 13; 29 g to 173 g) from healthy controls were mixed with a same fecal weight volume of PBS (Phosphate buffered saline, Lonza, Basel, Switzerland) using a vortex. The fecal suspension was centrifuged at 10,000× *g* for 30 min and the supernatant was centrifuged again at 30,000× *g* for 15 min. The supernatant was stored at −80 °C after vacuum filtration with a 0.22 µm filter (Steritop threaded bottle top filter, Merck Chemicals, Brussels, Belgium) to remove viable bacteria. The different media tested are summarized in Table S3. The bacterial test panel included *Bacteroides fragilis* (LMG 10263^T^), *B. thetaiotaomicron* (LMG 11262), *Bifidobacterium adolescentis* (LMG 10502^T^), *B. bifidum* (LMG 11041^T^), *B. longum* (LMG 13197^T^), *Clostridioides difficile* (LMG 21717^T^); *Clostridium sporogenes* (LMG 8421^T^ t1), *Lactobacillus acidophilus* (CHCC 3777), *L. paracasei* (LAB109cas), *Paraclostridium bifermentans* (LMG 3029), *Escherichia coli* (LMG 2092^T^) and *Staphylococcus epidermidis* (U9108901). Growth of the bacterial test panel in the broth media was determined spectrophotometrically at 600 nm (Glomax^®^ Explorer Multimode Microplate Reader, Promega, Madison, Wisconsin) and growth was also confirmed inoculating the broth onto SCH agar plates.

Furthermore, to select the culture media that enabled maximal recovery of different fecal bacteria, three fecal samples, from three controls, were cultured on five selected media, i.e., the only ones out of 28 combinations that supported growth of all species of the test panel. For this purpose, 1 g of each of the samples was homogenized and 0.5 g of the homogenate was used to make a tenfold serial dilution, using maximum recovery diluent solution (MRD, Sigma Aldrich). Fecal dilutions of 10^-2^ to 10^-4^ were used to inoculate agar plates for aerobic incubation (2 days at 37 °C), and fecal dilutions of 10^-4^ to 10^-6^ for anaerobic incubation (7 days at 37 °C) (Figure S2). The colony forming units (CFU) on each agar plate were counted and identified.

### 4.3. Isolation of Protein-Bound Uremic Toxin Precursor-Generating Bacteria from CKD Fecal Samples

Fecal samples were randomly selected from CKD 1 (*n* = 6) and CKD 5 (*n* = 6) patients and used to culture in vitro PBUT precursor-generating bacteria. First, a tenfold serial dilution of each fecal sample was prepared, with MRD as diluent solution. Fecal dilutions of 10^−2^ to 10^−4^ were used for aerobic incubation (2 days at 37 °C), and fecal dilutions of 10^−4^ to 10^−6^ for anaerobic incubation (6 days at 37 °C) were inoculated on YCFAG Δ0 +AAA +FS, SCH and 1203a media (CM0957 Anaerobe Basal Broth, Oxoid, Thermo Fisher Scientific, Merelbeke, Belgium), of which the last medium was recommended by the Deutsche Sammlung von Mikroorganismen und Zellkulturen GmbH (DSMZ) to culture *Akkermansia muciniphila*.

### 4.4. Identification of Fecal Protein-Bound Uremic Toxin Precursor-Generating Bacteria

Direct identification of bacterial colonies was performed with a matrix assisted laser desorption/ionization time-of-flight analyzer (MALDI-TOF, Bruker, Mannheim, Germany). For MALDI-TOF identification, the bacterial colonies were spotted on a MALDI-TOF plate and 1 µL of Bruker HCCA matrix solution (For 250 mL: 2.5 ± 0.3 mg α-Cyano-4-hydroxycinnamic acid, 125 mL acetonitrile, 118.75 mL water, and 6.25 mL trifluoroacetic acid) was added. In case of no identification with MALDI-TOF, 16S rRNA gene sequencing was performed by GATC (Eurofins Genomics, Ebersberg, Germany).

### 4.5. Quantification of p-Cresol, Phenol, Indole and Indole-3-Acetic Acid in Broth Media

The concentrations of *p*-cresol (108 Da) and indole (117 Da) in the broth medium were measured with reversed-phase HPLC. Chromatographic HPLC separation was performed at 26 °C on a reversed-phase XBridge C8 column (3.5 µm, 150 × 4.6 mm, Waters, Zellik, Belgium) with an Ultrasphere 5 ODS Guard column (5 µm, 45 × 4.6 mm, Hichrom, Reading, UK). The mobile phase consisted of 50 mM ammonium formate buffer (mobile phase A, pH 3.0) and methanol (mobile phase B). The chromatographic separation consisted of a linear gradient at a flow of 1 mL/min, starting with 100% A during the first 3 min, followed by a change into 100% B during the next 36 min. The latter composition was held for 3 min and followed by a re-equilibration step. Fecal suspensions were filtered before chromatography through an Amicon Ultra 0.5 mL filter (molecular weight cut-off 30 kDa, Millipore Merck, Darmstadt, Germany). *p*-Cresol (λ_ex_: 278 nm, λ_em_: 304 nm), and indole (λ_ex_: 275 nm, λ_em_: 334 nm) were detected by a Waters 2475 fluorescence detector.

Concentrations of phenol (94 Da) and IAA (175 Da) were measured with reversed-phase UPLC analysis. For the quantification of the total concentration of IAA, samples were deproteinized by heat denaturation, followed by a filtration step through an Amicon Ultra 0.5 mL filter prior to reversed-phase UPLC analysis [89]. Chromatographic UPLC separation was performed at 26 °C on a Waters Acquity UPLC BEH C18 Van Guard column (1.7 µm, 100 x 2.1 mm). The mobile phase consisted of 50 mM ammonium formate buffer (mobile phase A, pH 3.0) and methanol (mobile phase B). A linear gradient elution was used to separate the compounds at a flow rate of 0.3 mL/min, and started at 98% A, followed by a composition change to 90% A in 7 min. In the next 9 min, the mobile phase changed to 100% B and was held for 3 min. Finally, a re-equilibration step was performed. Phenol (λ_ex_: 275 nm, λ_em_: 350 nm) and IAA (λ_ex_: 280 nm, λ_em_: 350 nm) were detected by an Agilent G1316C fluorescence detector (Agilent, Diegem, Belgium).

### 4.6. Quantification of Bacterial Species in Fecal Samples

To perform qPCR assays, bacterial DNA was extracted from fecal samples using the PowerMicrobiome RNA isolation kit (Qiagen, Hilden, Germany) with the following modifications: the DNase steps (steps 12 to 16) were not performed, in order to extract DNA next to RNA. Furthermore, an additional heating step of 95 °C for 10 min after step 4 was added, to increase the DNA yield [90,91].

In addition to the total bacterial number also the abundance of 11 bacterial taxa in different stages of CKD and controls was assessed. Of these, *Bacteroides* spp., *Bifidobacterium* spp., *C. difficile*, *Enterobacteriaceae*, *E. coli*, *F. prausnitzii*, *Roseburia* spp., and *Streptococcus* spp. were selected based on their PBUT precursor-generating capacity, observed in the present study and in in vitro studies [9]. Moreover, *A. muciniphila*, *Bacteroides* spp., *F. prausnitzii*, *Lactobacillus* spp., *Roseburia* spp., and *Streptococcus* spp. belonged to the top 10 bacterial taxa with the largest differences in proportions between HD patients with high *p*CS/low IxS versus low *p*CS/high IxS serum levels, shown in a previous study of our group [58], for which reason these were included here. Finally, the abundance of a well-known SCFA-generator *Butyricicoccus* spp. [71] was also assessed.

To quantify the bacterial taxa from fecal samples, family-, genus- and species-specific qPCRs were developed and performed on the basis of previously described formats [71,91,92,93,94,95,96,97,98,99,100,101,102,103]. Primers and probes used are summarized in Table S4. The specificity of primers and probes was evaluated in silico using Nucleotide BLAST (basic local alignment search tool). Primer sequences and the amplicon sequence were further evaluated with the programs “OligoAnalyzer”, “mfold” and “DINAMelt” for homo- and hetero-dimers, sequence length, GC percentage, and melting temperature. All primers and the probe were purchased from Kaneka Eurogentec (Seraing, Belgium). To validate the specificity of the amplification of the primers and probes, qPCR amplification was performed using template DNA from different bacterial species (Table S5), with the LightCycler480 (LC480, Roche Life Science, Vilvoorde, Belgium). Table S4 summarizes the qPCR mixes and thermal cycling conditions for each qPCR. To verify the specificity of the qPCRs without the usage of a probe, melting curve analysis was performed. Of every qPCR, the limit of quantification (LOQ) and LOD was determined, as described by Forootan et al. [104] (Table S4). For all qPCR reactions, every sample was amplified in duplicate, standard dilution series were used as positive controls, and qPCR mixes without DNA were included as negative controls. Standard tenfold dilution series were prepared from DNA extracts indicated in Table S5.

### 4.7. Statistical Analysis

All statistical analyses were performed by IBM SPSS Statistics for Windows, version 26 (IBM, Armonk, NY, USA) and graphs were made with R (2019, version 3.6.0). Prior to statistical analysis of the qPCR concentration results of the bacterial species, the data were log_10_ transformed. To determine the normality of the data, a Shapiro-Wilk test was performed. Depending on the distribution, the data are presented as mean ± standard deviation or median (25th–75th percentile). To compare the characteristics and the bacterial concentrations between the different stages of CKD (control, CKD stage 1–5, HD and PD), an ANOVA or a Kruskal-Wallis test, both with a Benjamini-Hochberg correction for multiple testing, was performed according the data distribution. Correlations between the abundance of the bacterial taxa and different study groups (control and CKD 1-5) or eGFR, were performed with the Spearman’s rank test (*r_s_*). Differences were considered as statistically significant when the *p*-value was below 0.05.

## Figures and Tables

**Table 1:** Protein-bound uremic toxin precursor-generating capacity of bacteria isolated from fecal samples of patients with chronic kidney disease (CKD).

Bacterial Species	CKD 1 (*n* = 6)					CKD 5 (*n* = 6)				
	Neg	*p*C	Phe	Ind	IAA	Neg	*p*C	Phe	Ind	IAA
**ACTINOBACTERIA**										
***Actinomycetaceae***										
*Actinomyces sp.*							O^4^			O^4^
***Bifidobacteriaceae***										
*Bifidobacterium adolescentis*	N^9^					N^1^				
*Bifidobacterium bifidum ^c^*					N^1^					N^2^
*Bifidobacterium catenulatum/* *pseudocatenulatum ^a^*					N^9^					N^2^
*Bifidobacterium dentium*		N^1^			N^1^					
*Bifidobacterium longum ^c^*					N^6^					N^5^
*Parascardovia denticolens*						NC				
*Scardovia wiggsiae*										N^1^
***Brevibacteriaceae***										
*Brevibacterium ravenspurgense*										O^1^
***Coriobacteriaceae***										
*Collinsella aerofaciens*					O^1^					N^4^
***Corynebacteriaceae***										
*Corynebacterium amycolatum*					O^1^					O^1^
*Corynebacterium aurimucosum*	NC					NC				
*Corynebacterium coyleae*	O^3^									
***Eggerthellaceae***										
*Eggerthella lenta*										N^3^
***Microbacteriaceae***										
*Microbacterium sp.*					O^2^					
***Micrococcaceae***										
*Kocuria sp.*					O^1^					
*Pseudoglutamicibacter albus*					O^1^					
***Propionibacteriaceae***										
*Cutibacterium acnes*				N^2^	N^2^				N^1^	N^1^
*Propionibacterium freudenreichii*										N^1^
**BACTEROIDETES**										
***Bacteroidaceae***										
*Bacteroides sp.*		N^1^	N^1^		N^1^		N^1^			N^1^
*Bacteroides caccae*		N^1^	N^1^		N^1^		N^1^	N^1^		N^1^
*Bacteroides cellulosilyticus*				N^1^	N^1^					
*Bacteroides coprocola*							N^1^			N^1^
*Bacteroides eggerthii*			N^1^	N^1^	N^1^			N^1^	N^1^	N^1^
*Bacteroides faecis*									N^1^	N^1^
*Bacteroides finegoldii*		N^1^	N^1^		N^1^					
*Bacteroides fragilis*		N^1^	N^1^		N^1^		O^1^	O^1^		O^1^
*Bacteroides intestinalis*	NC									
*Bacteroides massiliensis*							N^1^			N^1^
*Bacteroides ovatus*		N^1^	N^1^		N^1^			N^1^		N^1^
*Bacteroides pyogenes*							N^1^			N^1^
*Bacteroides salyersiae*		N^1^			N^1^					
*Bacteroides stercoris*	NC									
*Bacteroides thetaiotaomicron*									N^2^	N^2^
*Bacteroides uniformis*		N^1^	N^1^	N^1^	N^1^		N^2^	N^2^	N^4^	N^3^
*Bacteroides vulgatus*		N^2^	N^1^		N^2^		N^1^			N^1^
***Odoribacteriaceae***										
*Odoribacter splanchnicus*							N^3^	N^3^	N^3^	N^3^
***Prevotellaceae***										
*Prevotella copri*			N^1^		N^1^					
***Rikenellaceae***										
*Alistipes putredinis*									N^1^	N^1^
*Alistipes shahii*				N^2^	N^2^					
***Tannerellaceae***										
*Parabacteroides distasonis*			N^2^		N^2^					
*Parabacteroides goldsteinii*			N^1^		N^1^					
*Parabacteroides johnsonii*			N^1^		N^1^					
*Parabacteroides merdae*							N^1^	N^2^		N^3^
**FUSOBACTERIA**										
***Fusobacteriaceae***										
*Fusobacterium sp.*							N^1^		N^1^	N^1^
**FIRMICUTES**										
***Acidaminococcaceae***										
*Acidaminococcus intestine*					N^1^					
***Bacillaceae***										
*Bacillus amyloliquefaciens*							N^1^		N^1^	N^1^
*Bacillus cereus*					O^5^					
*Bacillus licheniformis*					O^1^					
*Bacillus pumilus*					O^1^					
*Bacillus sonorensis*					O^1^					
*Bacillus subtilis*		O^1^			O^1^					
***Clostridiaceae***										
*Clostridium perfringens*							N^1^			N^1^
*Clostridium tertium*		O^1^			O^1^					
***Enterococcaceae***										
*Enterococcus avium*							O^1^			O^1^
*Enterococcus casseliflavus*		O^1^	O^1^		O^1^					
*Enterococcus durans ^d^*	O^2^									
*Enterococcus faecalis ^d^*					O^2^					O^2^
*Enterococcus faecium ^d^*	O^9^	N^1^	N^1^			O^3^	N^2^			
***Erysipelotrichaceae***										
*Coprobacillus cateniformis*						NC				
*Catenibacterium mitsuokai*						N^1^				
*Erysipelatoclostridium ramosum ^c^*						N^2^				
***Lachnospiraceae***										
*Lachnoclostridium scindens*							N^1^			N^1^
*Lachnoclostridium symbiosum ^c^*							N^1^	N^1^		N^1^
***Lactobacillaceae***										
*Lactobacillus buchneri*						NC				
*Lactobacillus delbrueckii ^c^*							N^1^			
*Lactobacillus paracasei*	NC					NC				
*Lactobacillus rhamnosus*					O^1^/N^1^					O^1^/N^1^
*Lactobacillus ruminis*	NC									
*Lactobacillus sakei*	NC									
*Pediococcus acidilactici ^c^*		O^1^			O^1^					
***Oscillospiraceae***										
*Oscillibacter sp.*		N^1^	N^1^	N^1^	N^1^					
***Ruminococcaceae***										
*Flavonifractor plautii*						NC				
***Staphylococcaceae***										
*Staphylococcus capitis*		N^1^			N^1^					
*Staphylococcus epidermidis*			N^1^		N^1^					
*Staphylococcus haemolyticus*								O^2^	O^2^	O^2^
*Staphylococcus hominis*						NC				
***Streptococcaceae***										
*Lactococcus lactis*						NC				
*Streptococcus agalactiae*						O^1^				
*Streptococcus anginosus*	NC									
*Streptococcus gordonii*								O^1^		O^1^
*Streptococcus lutetiensis*						O^1^/N^2^				
*Streptococcus oralis/mitis ^a^*								O^3^		
*Streptococcus parasanguinis*	NC									
*Streptococcus sanguinis/* *australis ^b^*					N^1^					N^1^
*Streptococcus vestibularis/* *salivarius ^a^*		O^1^			O^1^					O^3^
***Veillonellaceae***										
*Megasphaera elsdenii*	NC					NC				
*Veillonella atypica*	NC									
*Veillonella dispar*	NC									
**PROTEOBACTERIA**										
***Caulobacteraceae***										
*Brevundimonas diminuta*										O^1^
***Desulfovibrionaceae***										
*Bilophila wadsworthia*		N^1^			N^1^					
***Enterobacteriaceae***										
*Citrobacter freundii*									O^1^	O^1^
*Enterobacter cloacae/kobei/ludwigii* */xiangfangensis/hormaechei ^a^*					O^6^/N^1^					
Escherichia coli *				O^7^/N^1^	O^7^/N^1^				O^15^/N^4^	O^15^/N^4^
*Klebsiella variicola ^c^*				O^1^	O^1^					
***Pseudomonadaceae***										
*Pseudomonas aeruginosa*					O^1^					O^1^
*Pseudomonas stutzeri*										O^1^
***Sutterellaceae***										
*Sutterella wadsworthensis*	NC									
***Xanthomonodaceae***										
*Stenotrophomonas maltophilia*						NC				
**NO IDENTIFICATION°**										
*MB 208_49*							N^1^	N^1^	N^1^	N^1^
*MB 210_6*					N^1^					
*MB 210_38*			N^1^		N^1^					
*MB 242_16*										N^1^
*MB 256_36*					N^1^					
*MB 256_39*			N^1^		N^1^					
*MB 256_61*		N^1^	N^1^		N^1^					
*MB 263_32*					N^1^					
*MB 275_15*							N^1^			N^1^
*MB 275_16*										N^1^
*MB 300_21*		N^1^			N^1^					
*MB 314_01*				O^1^	O^1^					

Bacterial taxonomy according to Uniprot [50]; ^a^: species not distinguishable with MALDI-TOF; ^b^: species not distinguishable with 16S rRNA gene sequencing; ^c^: species with generation of unknown peak 26 on HPLC; ^d^: species with generation of an unknown peak 9 on HPLC; °: no identification after MALDI-TOF and/or 16S rRNA gene sequencing; *: not distinguishable with *Shigella* spp. with MALDI-TOF; Neg.: No in vitro PBUT precursor generation capacity; *p*C: *p*-cresol; Phe: phenol; Ind: indole; IAA: indole-3-acetic acid; O (oxygen): in vitro PBUT precursor generation capacity under aerobic conditions (37 °C for 2 days); N (nitrogen): in vitro PBUT precursor generation capacity under anaerobic conditions (37 °C for 6 days); x (in O^x^/N^x^): Number of isolates tested for uremic toxin precursor generation; NC: could not be further cultured in broth to check the generation capacity of PBUT precursors.

## Supplementary Materials

Supplementary materials can be found at https://www.mdpi.com/1422-0067/21/6/1986/s1. Figure S1: Ability of the 12 bacterial species of the test panel to grow on 28 different media, Figure S2: Isolation and identification of protein-bound uremic toxin (PBUT) precursor-generating bacteria; Table S1: Isolated organisms from three fecal samples on five different media; Table S2: Correlation between the abundance of the bacterial taxa and estimated glomerular filtration rate (eGFR); Table S3: Media composition (for 1L), Table S4: Primers, probes and thermal cycling conditions for all performed qPCRs, Table S5: qPCR specificity.

## Abbreviations

AAA	Aromatic amino acids
CKD	Chronic kidney disease
eGFR	Estimated glomerular filtration rate
ESKD	End stage kidney disease
FS	Fecal suspension
GOS	Galactooligosaccharides
HD	Hemodialysis
IAA	Indole-3-acetic acid
IN	Enriched inulin
IxS	Indoxyl sulfate
LOD	Limit of detection
LOQ	Limit of quantification
OF	Oligofructose
PBUT	Protein-bound uremic toxin
*p*CS	*p*-Cresyl sulfate
pCG	*p*-Cresyl glucuronide
PD	Peritoneal dialysis
SCFA	Short chain fatty acid
SCH	Schaedler medium
YCFAG	Yeast Casitone Fatty Acid Glucose medium
