# Supplementary material for: Isolation and Quantification of Uremic Toxin Precursor-Generating Gut Bacteria in Chronic Kidney Disease Patients
Source: Int J Mol Sci. 2020 Mar 14;21(6):1986. doi: 10.3390/ijms21061986 (PMC7139965; doi:10.3390/ijms21061986)
Supplement: Supplementary file 1 [file ijms-21-01986-s001.pdf]

## SUPPLEMENTARY TABLES AND FIGURES

**Table S1:** Isolated microorganisms from three fecal samples from controls on five different media.

| | YCFAG | | | SCH | |
|---|---|---|---|---|---|
| | Δ0 +AAA +FS | ΔV +AAA +FS | ΔS +AAA +FS | SCH | SCH +FS |
| **ACTINOBACTERIA** | | | | | |
| ***Bifidobacteriaceae*** | | | | | |
| *Bifidobacterium catenulatum/ pseudocatenulatum* | N | N | N | N | N |
| *Bifidobacterium longum* | N | N | N | N | |
| ***Coriobacteriaceae*** | | | | | |
| *Collinsella aerofaciens* | N | | | | |
| ***Corynebacteriaceae*** | | | | | |
| *Corynebacterium sp.* | O | O | | | O |
| *Corynebacterium aurimucosum* | | O | O | | |
| *Corynebacterium striatum* | | O | O | | |
| **BACTEROIDETES** | | | | | |
| ***Bacteroideaceae*** | | | | | |
| *Bacteroides caccae* | | N | | | |
| *Bacteroides cellulosilyticus* | | N | N | | |
| *Bacteroides fragilis* | N | | | | |
| *Bacteroides ovatus* | N | | | N | |
| *Bacteroides thetaiotaomicron* | | | N | | |
| *Bacteroides uniformis* | | N | | N | |
| *Bacteroides vulgatus* | N | N | N | N | |
| ***Tannerellaceae*** | | | | | |
| *Parabacteroides distasonis* | N | | | | |
| **FIRMICUTES** | | | | | |
| ***Bacillaceae*** | | | | | |
| *Bacillus sp.* | | | O | | |
| *Bacillus pumilus* | O | O | | | |
| ***Enterococcaceae*** | | | | | |
| *Enterococcus faecium* | O/N | O/N | O/N | O | O/N |
| *Enterococcus mundtii* | O | | O | | O |
| ***Erysipelotrichaceae*** | | | | | |
| *Clostridium innocuum* | | N | | N | |
| ***Staphylococcaceae*** | | | | | |
| *Staphylococcus sp.* | | | | | O |
| *Staphylococcus parasanguinis* | O | | | | |
| **PROTEOBACTERIA** | | | | | |
| ***Enterobacteriaceae*** | | | | | |
| *Citrobacter amalonaticus* | | | | O | |
| *Escherichia coli* | O/N | O/N | O/N | O/N | O/N |
| **EUKARYOTES** | | | | | |
| *Candida albicans* | | | | O | |
| **TOTAL N° OF DIFFERENT ISOLATES (O/N)** | **6/9** | **6/9** | **6/7** | **4/7** | **5/3** |

YCFAG: Yeast Casitone Fatty Acid Glucose medium; Δ0: medium unchanged; ΔV: medium without vitamins; ΔS: medium without short chain fatty acids; FS: fecal suspension; AAA: aromatic amino acids; SCH: Schaedler medium; O: isolated from fecal samples on corresponding medium after aerobic incubation (37 °C for 2 to 5

days); N: isolated from fecal samples on corresponding medium after anaerobic incubation (37 °C for 5 to 7 days).

**Table S2:** Correlation between the abundance of the bacterial taxa, as assessed by means of qPCR, and estimated glomerular filtration rate (eGFR).

| | **eGFR** | |
|---|---|---|
| | $r_s$ | ***p*-value** |
| Total number of bacterial 16S rRNA gene copies | 0.178 | 0.053 |
| *Akkermansia muciniphila* | 0.131 | 0.147 |
| *Bacteroides* spp. | 0.118 | 0.193 |
| *Bifidobacterium* spp. | 0.255 | 0.004 |
| *Butyricicoccus* spp. | 0.328 | <0.001 |
| *Clostridioides difficile* | -0.137 | 0.129 |
| *Enterobacteriaceae* | -0.201 | 0.027 |
| *Escherichia coli* | -0.252 | 0.005 |
| *Faecalibacterium prausnitzii* | 0.220 | 0.014 |
| *Lactobacillus* spp. | 0.137 | 0.131 |
| *Roseburia* spp. | 0.324 | <0.001 |
| *Streptocococcus* spp. | 0.143 | 0.127 |

$r_s$: Spearman's rank correlation coefficient

**Table S3:** Media composition (for 1 L)

| Products | YCFAG +AAA+FS | YCFAG ΔG +AAA +FS | YCFAG ΔC +AAA +FS | YCFAG ΔM +AAA +FS | YCFAG ΔV +AAA +FS | YCFAG ΔS +AAA +FS | SCH +AAA +FS | 1203a |
|---|---|---|---|---|---|---|---|---|
| Anaerobe basal broth (Thermo Fisher Scientific) (g) | N/A | N/A | N/A | N/A | N/A | N/A | N/A | 35.4 |
| Bacto™ agar (BD)° (g) | 15.0 | 15.0 | 15.0 | 15.0 | 15.0 | 15.0 | 15.0 | 15.0 |
| Bacto™ Casitone (BD) (g) | 10.0 | 10.0 | N/A | 10.0 | 10.0 | 10.0 | N/A | N/A |
| Fecal suspension, sterile* (ml) | 100.0 | 100.0 | 100.0 | 100.0 | 100.0 | 100.0 | 100.0 | N/A |
| Glucose (g) | 4.50 | N/A | 4.50 | 4.50 | 4.50 | 4.50 | N/A | N/A |
| L-Cystein-HCl* (g) | 1.0 | 1.0 | 1.0 | 1.0 | 1.0 | 1.0 | N/A | N/A |
| L-Phenylalanine* (mg) | 413.0 | 413.0 | 413.0 | 413.0 | 413.0 | 413.0 | 413.0 | N/A |
| L-Tryptophan* (mg) | 511.0 | 511.0 | 511.0 | 511.0 | 511.0 | 511.0 | 511.0 | N/A |
| L-Tyrosine* (mg) | 453.0 | 453.0 | 453.0 | 453.0 | 453.0 | 453.0 | 453.0 | N/A |
| $NaHCO^{3}$* (g) | 4.0 | 4.0 | 4.0 | 4.0 | 4.0 | 4.0 | N/A | N/A |
| Resazurin (of 1 mg/ml) (ml) | 1.0 | 1.0 | 1.0 | 1.0 | 1.0 | 1.0 | N/A | N/A |
| SCH anaerobe broth (g) | N/A | N/A | N/A | N/A | N/A | N/A | 26.4 | N/A |
| Yeast extract (g) | 2.5 | 2.5 | 2.5 | 2.5 | 2.5 | 2.5 | N/A | N/A |
| **Minerals** | | | | | | | | |
| $CaCl_2$ (mg) | 90.0 | 90.0 | 90.0 | N/A | 90.0 | 90.0 | N/A | N/A |
| $K_2HPO_4$ (mg) | 450.0 | 450.0 | 450.0 | N/A | 450.0 | 450.0 | N/A | N/A |
| $KH_2PO_4$ (mg) | 450.0 | 450.0 | 450.0 | N/A | 450.0 | 450.0 | N/A | N/A |
| $MgSO_4.7H_2O$ (mg) | 90.0 | 90.0 | 90.0 | N/A | 90.0 | 90.0 | N/A | N/A |
| NaCl (mg) | 900.0 | 900.0 | 900.0 | N/A | 900.0 | 900.0 | N/A | N/A |
| **Vitamins – Growth factors** | | | | | | | | |
| Biotin* (µg) | 10.0 | 10.0 | 10.0 | 10.0 | N/A | 10.0 | N/A | N/A |
| Cobalamin* (µg) | 10.0 | 10.0 | 10.0 | 10.0 | N/A | 10.0 | N/A | N/A |
| Haemin (mg) | 10.0 | 10.0 | 10.0 | 10.0 | N/A | 10.0 | N/A | N/A |
| Folic acid* (µg) | 50.0 | 50.0 | 50.0 | 50.0 | N/A | 50.0 | N/A | N/A |
| *p*-aminobenzoic acid* (µg) | 30.0 | 30.0 | 30.0 | 30.0 | N/A | 30.0 | N/A | N/A |
| Pydidoxine-HCl* (µg) | 150.0 | 150.0 | 150.0 | 150.0 | N/A | 150.0 | N/A | N/A |
| Riboflavin* (µg) | 50.0 | 50.0 | 50.0 | 50.0 | N/A | 50.0 | N/A | N/A |
| Thiamin* (µg) | 50.0 | 50.0 | 50.0 | 50.0 | N/A | 50.0 | N/A | N/A |
| **Short chain fatty acids** | | | | | | | | |
| Acetate (ml) | 1.948 | 1.948 | 1.948 | 1.948 | 1.948 | N/A | N/A | N/A |

| | | | | | | | | |
|---|---|---|---|---|---|---|---|---|
| Propionate (ml) | 0.658 | 0.658 | 0.658 | 0.658 | 0.658 | N/A | N/A | N/A |
| Isobutyrate (ml) | 0.088 | 0.088 | 0.088 | 0.088 | 0.088 | N/A | N/A | N/A |
| Isovalerate (ml) | 1.021 | 1.021 | 1.021 | 1.021 | 1.021 | N/A | N/A | N/A |
| Valerate (ml) | 1.021 | 1.021 | 1.021 | 1.021 | 1.021 | N/A | N/A | N/A |
| pH | 6.8 ±0.1 | 6.8 ±0.1 | 6.8 ±0.1 | 6.8 ±0.1 | 6.8 ±0.1 | 6.8 ±0.1 | 6.8 ±0.1 | 6.8 ±0.1 |

YCFAG: Yeast Casitone Fatty Acid Glucose; SCH: Schaedler medium; AAA: aromatic amino acids (L-phenylalanine, L-tyrosine, and L-tryptophan); FS: fecal suspension; ΔG: medium without glucose; ΔC: medium without casitone; ΔM: medium without minerals; ΔV: medium without vitamins; ΔS: medium without short chain fatty acids; BD: Becton Dickinson; N/A: not applicable; *: added after autoclaving; °: added to prepare agar plates; all products were purchased from Sigma Aldrich unless stated otherwise.

**Table S4:** Primers, probes and thermal cycling conditions for all performed qPCRs

| qPCR<br>(Target gene) | Pos/<br>Neg<br>(%)° | Sequence | length<br>(bp) | Primer/<br>Probe<br>(µM) | $MgCl_2$<br>(mM) | Thermal cycling conditions | | | | LOQ<br>LOD<br>($log_{10}$) | Ref. |
|---|---|---|---|---|---|---|---|---|---|---|---|
| | | | | | | Pre-step | Amplification | Melting | Cooling | | |
| **Total bacterial 16S rRNA gene copies**<br>(16S rRNA gene) | 96/0 | F: ACTCCTACGGGAGGCAGCAGT<br>R: GTAATTCCGCGGCTGCTGGCAC | 194-200 | 0.5<br>0.5 | 3 | 10′<br>95°C | 45x 15″ 95°C<br>40″ 60°C<br>30″ 72°C | 5″ 95°C<br>1′ 55°C<br>97°C* | 30″<br>40°C | 5.04<br>4.80 | [1] |
| ***Akkermansia muciniphila***<br>(16S rRNA gene) | 89/11 | F: CAGCACGTGAAGGTGGGGAC<br>R: CCTTGCGGTTGGCTTCAGAT | 329 | 0.5<br>0.5 | 2 | 10′<br>95°C | 40x 15″ 95°C<br>40″ 64°C<br>30″ 72°C | 5″ 95°C<br>1′ 60°C<br>97°C* | 30″<br>40°C | 4.11<br>3.53 | [1, 2] |
| ***Bacteroides* spp.**<br>(16S rRNA gene) | 100/0 | F: GGGTTTAAAGGGAGCGTAGG<br>R: CTACACCACGAATTCCGCCT<br>P: FAM[a]-TAAGTCAGTTGTGAAAGTTTGCGGCTC-TAMRA[b] | 116 | 0.3<br>0.3<br>0.2 | 0 | 10′<br>95°C | 45x 30″ 95°C<br>45″ 60°C | N/A | 30″<br>40°C | 3.32<br>2.75 | [3, 4] |
| ***Bifidobacterium* spp.**<br>(16S rRNA gene) | 100/0 | F: GAATAGCTCCTGGAAACG<br>R: ATAGGACGCGACCCCA<br>P: FAM[a]-TGGTAATGCCGGATGCTCC-TAMRA[b] | 99 | 0.3<br>0.3<br>0.2 | 0 | 10′<br>95°C | 45x 15″ 95°C<br>60″ 62°C | N/A | 30″<br>40°C | 2.69<br>1.75 | Based on [4] |
| ***Butyricicoccus* spp.**<br>(16S rRNA gene) | 100/0 | F: ACCTGAAGAATAAGCTCC<br>R: GATAACGCTTGCTCCCTACGT | 69 | 0.5<br>0.5 | 3 | 10′<br>95°C | 45x 15″ 95°C<br>40″ 58°C<br>30″ 72°C | 5″ 95°C<br>1′ 55°C<br>97°C* | 30″<br>40°C | 2.59<br>2.51 | [5] |
| ***Clostridioides difficile***<br>(16S rRNA gene) | 9/91 | F: GCAAGTTGAGCGATTTACTTCGGT<br>R: GTACTGGCTCACCTTTGATATTYAAGAG<br>P: FAM[a]-TGCCTCTCAAATATATTATCCCGTATTAG-TAMRA[b] | 155 | 0.2<br>0.2<br>0.2 | 0 | 10′<br>95°C | 45x 5″ 95°C<br>50″ 56°C | N/A | 30″<br>40°C | 3.26<br>2.27 | [6] |
| ***Enterobacteriaceae* spp.**<br>(16S rRNA gene) | 97/0 | F: CATTGACGTTACCCGCAGAAGAAGC<br>R: CTCTACGAGACTCAAGCTTGC | 190 | 0.5<br>0.5 | 3 | 10′<br>95°C | 45x 15″ 95°C<br>40″ 63°C<br>30″ 72°C | 5″ 95°C<br>1′ 55°C<br>97°C* | 30″<br>40°C | 3.50<br>3.47 | [1, 7] |
| ***Escherichia coli***<br>(*uidA* gene) | 97/3 | F: CAACGAACTGAACTGGCAGA<br>R: CATTACGCTGCGATGGAT<br>P: FAM[a]-TATCCCGCCGGGAATGGTGA-TAMRA[b] | 121 | 0.3<br>0.3<br>0.2 | 0 | 10′<br>95°C | 45x 15″ 95°C<br>60″ 64°C | N/A | 30″<br>40°C | 4.67<br>4.20 | [8, 9] |
| ***Faecalibacterium prausnitzii***<br>(16S rRNA gene) | 100/0 | F: GGAGGAAGAAGGTCTTCGG<br>R: AATTCCGCCTACCTCTGCACT | 248 | 0.5<br>0.5 | 3 | 10′<br>95°C | 45x 15″ 95°C<br>40″ 60°C<br>30″ 72°C | 5″ 95°C<br>1′ 55°C<br>97°C* | 30″<br>40°C | 3.66<br>2.43 | [10, 11] |
| ***Lactobacillus* spp.**<br>(16S rRNA gene) | 90/9 | F: AGCAGTAGGGAATCTTCCA<br>R: CACCGCTACACATGGAG | 340-346 | 0.3<br>0.3 | 2 | 10′<br>95°C | 45x 15″ 95°C<br>40″ 58°C<br>30″ 72°C | 5″ 95°C<br>1′ 55°C<br>97°C* | 30″<br>40°C | 6.05<br>4.12 | [12] |
| ***Roseburia* spp.**<br>(16S rRNA gene) | 99/0 | F: TACTGCATTGGAAACTG<br>R: CGGCACCGAAGAGCAAT | 230 | 0.5<br>0.5 | 3 | 10′<br>95°C | 45x 15″ 95°C<br>40″ 60°C<br>30″ 72°C | 5″ 95°C<br>1′ 55°C<br>97°C* | 30″<br>40°C | 2.37<br>2.22 | [13] |
| ***Streptococcus* spp.**<br>(16S rRNA gene) | 81/12 | F: GAAGAATTGCTTGAATTGGTTGAA<br>R: GGACGGTAGTTGTTGAAGAATGG | 559 | 0.5<br>0.5 | 3 | 10′<br>95°C | 45x 15″ 95°C<br>40″ 60°C<br>30″ 72°C | 5″ 95°C<br>1′ 55°C<br>97°C* | 30″<br>40°C | 5.11<br>4.45 | [14] |

F: forward primer sequence; R: reverse primer sequence; P: probe; bp: basepair; Ref.: reference; a: 6-carboxyfluorescein, fluorescence reporter dye; b: 5(6)-carboxy-tetramethylrhodamine, fluorescence quencher dye; *: continuous rate at 0.02°C/sec; °: percentage of positive and negative qPCR signals; N/A: not applicable.

**Table S5:** qPCR specificity

| Bacterial species test panel (Origin) | TOT BACT | AKKMUC | BAT spp. | BIF spp. | BUT spp. | CLSDIF | ENT spp. | ESCCOL | FAEPRA | LAB spp. | ROS spp. | STR spp. |
|---|---|---|---|---|---|---|---|---|---|---|---|---|
| **ACTINOBACTERIA** | | | | | | | | | | | | |
| ***Bifidobacteriaceae*** | | | | | | | | | | | | |
| *Bifidobacterium animalis* (LBR 011205) | + | - | - | + | - | - | - | - | - | - | - | - |
| *Bifidobacterium bifidum* (LMG 11041$^{T}$) | + | - | - | + | - | - | - | - | - | - | - | - |
| *Bifidobacterium breve* (LBR 070975) | + | - | - | + | - | - | - | - | - | - | - | - |
| *Bifidobacterium catenulatum* (LBR 0715165) | + | - | - | + | - | - | - | - | - | - | - | - |
| *Bifidobacterium dentium* (LBR 710466) | + | - | - | + | - | - | - | - | - | - | - | - |
| *Bifidobacterium longum* (LMG 13197T) | + | - | - | + | - | - | - | - | - | - | - | - |
| *Bifidobacterium pseudocatenulatum* (LBR 0715206) | + | - | - | + | - | - | - | - | - | - | - | - |
| **BACILLUS** | | | | | | | | | | | | |
| ***Enterococcaceae*** | | | | | | | | | | | | |
| *Enterococcus durans* (LBR 0715214) | + | - | - | - | - | - | - | - | - | - | - | - |
| *Enterococcus faecalis* (LBR 0715156) | + | - | - | - | - | - | - | - | - | - | - | - |
| *Enterococcus faecalis* (LBR 0715102) | + | - | - | - | - | - | - | - | - | - | - | - |
| *Enterococcus faecium* (LBR 0715104) | + | - | - | - | - | - | - | - | - | - | - | - |
| ***Lactobacillaceae*** | | | | | | | | | | | | |
| *Lactobacillus acidophilus* (LBR 011207) | + | - | - | - | - | - | - | - | - | + | - | - |
| *Lactobacillus buchneri* (LBR 0715175) | + | - | - | - | - | - | - | - | - | + | - | - |
| *Lactobacillus fermentum* (0708283) | + | - | - | - | - | - | - | - | - | + | - | - |
| *Lactobacillus gasseri* (LBR 070934) | + | - | - | - | - | - | - | - | - | + | - | - |
| *Lactobacillus jensenii* (LMG 6414$^{T}$) | + | - | - | - | - | - | - | - | - | + | - | - |
| *Lactobacillus paracasei* (LBR 130272) | + | - | - | - | - | - | - | - | - | + | - | - |
| *Lactobacillus rhamnosus (LBR 020586)* | + | - | - | - | - | - | - | - | - | + | - | - |
| **BACTEROIDETES** | | | | | | | | | | | | |
| ***Bacteroidaceae*** | | | | | | | | | | | | |

| | | | | | | | | | | | | |
|---|---|---|---|---|---|---|---|---|---|---|---|---|
| *Bacteroides cellulolyticus* (LBR 0715207) | + | - | + | - | - | - | - | - | - | - | - | - |
| *Bacteroides fragilis* (LMG 10263$^T$) | + | - | + | - | - | - | - | - | - | - | - | - |
| *Bacteroides massieliensis* (LBR 0715233) | + | - | + | - | - | - | - | - | - | - | - | - |
| *Bacteroides ovatus* (LBR 0715138) | + | - | + | - | - | - | - | - | - | - | - | - |
| *Bacteroides thetaiotaomicron* (LMG 11262) | + | - | + | - | - | - | - | - | - | - | - | - |
| *Bacteroides thetaiotaomicron* (LBR 0715166) | + | - | + | - | - | - | - | - | - | - | - | - |
| *Bacteroides vulgatus* (LBR 0715135) | + | - | + | - | - | - | - | - | - | - | - | - |
| ***Tannerellaceae*** | | | | | | | | | | | | |
| *Parabacteroides goldsteinii* (LBR 0715235) | + | - | - | - | - | - | - | - | - | - | - | - |
| **FIRMICUTES** | | | | | | | | | | | | |
| ***Clostridiaceae*** | | | | | | | | | | | | |
| *Butyricicoccus faecihominis* (DSMZ 100989) | + | - | - | - | + | - | - | - | +* | - | - | - |
| *Butyricicoccus pullicaecorum* (DSMZ 23266) | + | - | - | - | + | - | - | - | +* | - | - | - |
| *Clostridium sporogenes* (LMG 8421$^T$) | + | - | - | - | - | - | - | - | - | - | - | - |
| *Clostridium tertium* (LBR 0715167) | + | - | - | - | - | - | - | - | - | - | - | - |
| ***Lachnospiraceae*** | | | | | | | | | | | | |
| *Roseburia hominis* (DSM 16839) | + | - | - | - | - | - | - | - | - | - | + | - |
| *Roseburia faecis* (DSM 16840) | + | - | - | - | - | - | - | - | - | - | + | - |
| ***Peptostreptococcaceae*** | | | | | | | | | | | | |
| *Clostridioides difficile* (LBR 011023) | + | - | - | - | - | + | - | - | - | - | - | - |
| *Clostridioides difficile* (LMG 21717$^T$) | + | - | - | - | - | + | - | - | - | - | - | - |
| *Paraclostridium bifermentans* (LMG 3029) | + | - | - | - | - | - | - | - | - | - | - | - |
| ***Ruminococcaceae*** | | | | | | | | | | | | |
| *Faecalibacterium prausnitzii* (DSMZ 17677) | + | - | - | - | - | - | - | - | + | - | - | - |
| *Faecalibacterium prausnitzii* (LBR M21/1) | + | - | - | - | - | - | - | - | + | - | - | - |
| ***Staphylococcaceae*** | | | | | | | | | | | | |
| *Staphylococcus hominis* (LBR 071563) | + | - | - | - | - | - | - | - | - | - | - | - |

| | | | | | | | | | | | | |
|---|---|---|---|---|---|---|---|---|---|---|---|---|
| ***Streptococcaceae*** | | | | | | | | | | | | |
| *Streptococcus mutans* (LMG 14558$^T$) | + | - | - | - | - | - | - | - | - | - | - | + |
| *Streptococcus parasanguinis* (LBR 0715158) | + | - | - | - | - | - | - | - | - | - | - | + |
| *Streptococcus pneumoniae* (LMG 14545$^T$) | + | - | - | - | - | - | - | - | - | - | - | + |
| *Streptococcus salivarius* (LBR 0715157) | + | - | - | - | - | - | - | - | - | - | - | + |
| **PROTEOBACTERIA** | | | | | | | | | | | | |
| ***Enterobacteriaceae*** | | | | | | | | | | | | |
| *Citrobacter freundii* (LBR 010201) | + | - | - | - | - | - | + | - | - | - | - | - |
| *Enterobacter cloacae* (LBR 0715229) | + | - | - | - | - | - | + | - | - | - | - | - |
| *Escherichia coli* (LMG 2092$^T$) | + | - | - | - | - | - | + | + | - | - | - | - |
| *Escherichia coli* (ATCC 25922) | + | - | - | - | - | - | + | + | - | - | - | - |
| *Escherichia fergusonii* (LBR 70436) | + | - | - | - | - | - | + | - | - | - | - | - |
| *Escherichia fergusonii* (LBR 111141) | + | - | - | - | - | - | + | - | - | - | - | - |
| *Escherichia hermanii* (LBR 130262) | + | - | - | - | - | - | + | - | - | - | - | - |
| *Escherichia hermanii* (LBR 111137) | + | - | - | - | - | - | + | - | - | - | - | - |
| *Klebsiella oxytoca* (LBR 010202) | + | - | - | - | - | - | + | - | - | - | - | - |
| *Pseudescherichia vulneris* (LBR 111139) | + | - | - | - | - | - | + | - | - | - | - | - |
| *Shigella boydii* (LBR 010146) | + | - | - | - | - | - | + | + | - | - | - | - |
| *Shigella boydii* (LBR 010152) | + | - | - | - | - | - | + | + | - | - | - | - |
| *Shigella flexneri* (LBR 070578) | + | - | - | - | - | - | + | + | - | - | - | - |
| *Shigella flexneri* (LBR 220544) | + | - | - | - | - | - | + | + | - | - | - | - |
| *Shigella sonnei* (LBR 170413) | + | - | - | - | - | - | + | + | - | - | - | - |
| ***Yersiniaceae*** | | | | | | | | | | | | |
| *Yersinia enterocolitica* (LBR 070622) | + | - | - | - | - | - | + | - | - | - | - | - |
| VERRUCOMICROBIA | | | | | | | | | | | | |
| ***Akkermansiaceae*** | | | | | | | | | | | | |
| *Akkermansia muciniphila* (DSM 22959) | + | + | - | - | - | - | - | - | - | - | - | - |

TOT BACT: Total bacteria; AKKMUC: *Akkermansia muciniphila*; BAT spp.: *Bacteroides* spp.; BIF spp.: *Bifidobacterium* spp.; BUT spp.; *Butyricicoccus* spp.: CLSDIF: *Clostridioides difficile*; ENT spp.: *Enterobacteriaceae* spp.; ENC spp.: *Enterococcus* spp.; ESCCOL: *Escherichia coli*; FAEPRA: *Faecalibacterium prausnitzii*; LACT spp.: *Lactobacillus* spp.; ROS spp.: *Roseburia* spp.; STR spp.: *Streptococcus* spp.; +: qPCR amplification; -: no qPCR amplification; gray: expected to be positive; *: cycle of amplification below limit of detection; bacterial strain underlined: strain used to prepare a standard tenfold dilution series.

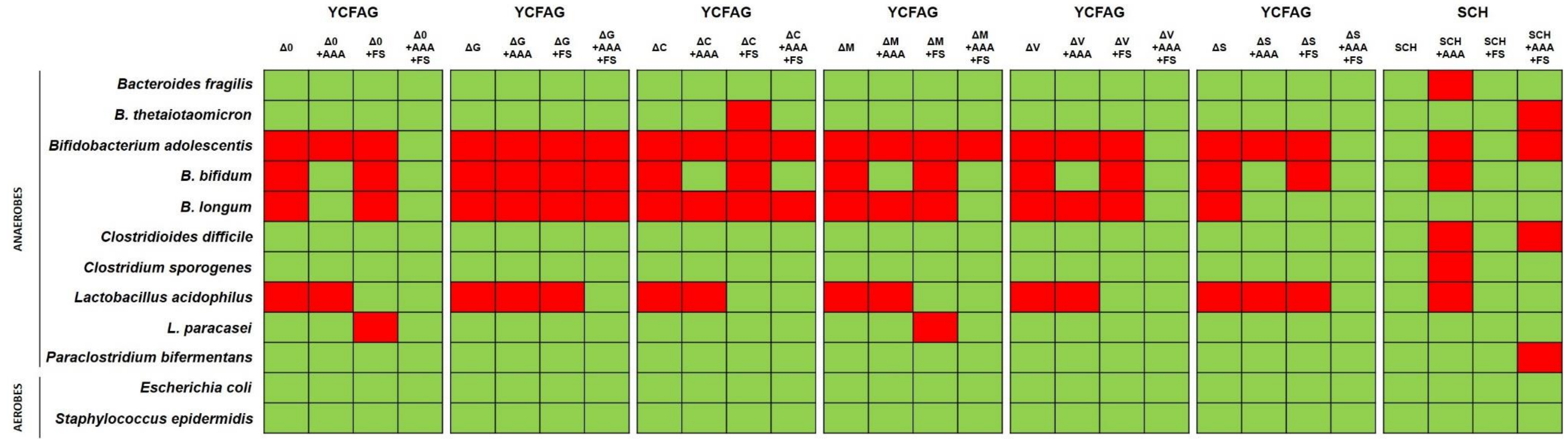


**Figure S1.** Ability of the 12 bacterial species of the test panel to grow on 28 different media. Aerobic incubation at 37 °C for 2 days and anaerobic incubation at 37 °C for 7 days; Red: no growth; Green: growth; YCFAG: Yeast Casitone Fatty Acid Glucose medium; Δ0: medium unchanged; ΔG: medium without glucose; ΔC: medium without casitone; ΔM: medium without minerals; ΔV: medium without vitamins; ΔS: medium without short chain fatty acids; SCH: Schaedler medium; FS: fecal suspension; AAA: aromatic amino acids.

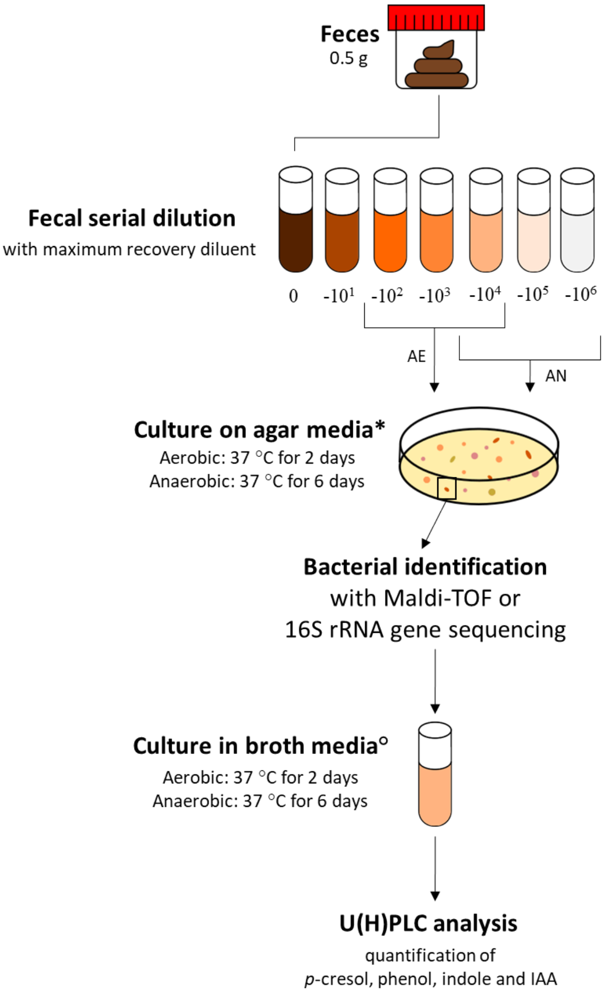


**FIGURE S2.** Isolation and identification of protein-bound uremic toxin (PBUT) precursor-generating bacteria. AE: aerobic: AN: anaerobic; IAA: indole-3-acetic acid; HPLC: High performance liquid chromatography; UPLC: Ultra performance liquid chromatography, *: three different media used: 1) Yeast Casitone Fatty Acid Glucose medium supplemented with aromatic amino acids and sterile fecal suspension, 2) Schaedler medium, and 3) 1203a medium; °: Yeast Casitone Fatty Acid Glucose broth medium supplemented with aromatic amino acids.

**Supplementary References**


1. Bergstrom, A.; Licht, T. R.; Wilcks, A.; Andersen, J. B.; Schmidt, L. R.; Gronlund, H. A.; Vigsnaes, L. K.; Michaelsen, K. F.; Bahl, M. I. Introducing GUt Low-Density Array (GULDA): a validated approach for qPCR-based intestinal microbial community analysis. *FEMS Microbiol Lett* **2012,** *337*, 38-47.
2. Collado, M. C.; Derrien, M.; Isolauri, E.; de Vos, W. M.; Salminen, S. Intestinal integrity and *Akkermansia muciniphila*, a mucin-degrading member of the intestinal microbiota present in infants, adults, and the elderly. *Appl Environ Microbiol* **2007,** *73*, 7767-7770.
3. Layton, A.; McKay, L.; Williams, D.; Garrett, V.; Gentry, R.; Sayler, G. Development of *Bacteroides* 16S rRNA gene TaqMan-based real-time PCR assays for estimation of total, human, and bovine fecal pollution in water. *Appl Environ Microbiol* **2006,** *72*, 4214-4224.
4. Hauther, K. A.; Cobaugh, K. L.; Jantz, L. M.; Sparer, T. E.; DeBruyn, J. M. Estimating time since death from postmortem human gut microbial communities. *J Forensic Sci* **2015,** *60*, 1234-1240.
5. Eeckhaut, V.; Machiels, K.; Perrier, C.; Romero, C.; Maes, S.; Flahou, B.; Steppe, M.; Haesebrouck, F.; Sas, B.; Ducatelle, R.; Vermeire, S.; Van Immerseel, F. *Butyricicoccus pullicaecorum* in inflammatory bowel disease. *Gut* **2013,** *62*, 1745-1752.
6. Kubota, H.; Sakai, T.; Gawad, A.; Makino, H.; Akiyama, T.; Ishikawa, E.; Oishi, K., Development of TaqMan-based quantitative PCR for sensitive and selective detection of toxigenic *Clostridium difficile* in human stools. *PloS one* **2014,** *9*, e111684.
7. Bartosch, S.; Fite, A.; Macfarlane, G. T.; McMurdo, M. E. Characterization of bacterial communities in feces from healthy elderly volunteers and hospitalized elderly patients by using real-time PCR and effects of antibiotic treatment on the fecal microbiota. *Appl Environ Microbiol* **2004,** *70*, 3575-3581.
8. Chern, E. C.; Siefring, S.; Paar, J.; Doolittle, M.; Haugland, R. A. Comparison of quantitative PCR assays for *Escherichia coli* targeting ribosomal RNA and single copy genes. *Lett Appl Microbiol* **2011,** *52*, 298-306.
9. Gryp, T.; Glorieux, G.; Joossens, M.; Vaneechoutte, M. Comparison of five assays for DNA extraction from bacterial cells in human faecal samples. *J Appl Microbiol* **2020**. doi: 10.1111/jam.14608.
10. Ramirez-Farias, C.; Slezak, K.; Fuller, Z.; Duncan, A.; Holtrop, G.; Louis, P. Effect of inulin on the human gut microbiota: stimulation of *Bifidobacterium adolescentis* and *Faecalibacterium prausnitzii*. *Br J Nutr* **2009,** *101*, 541-550.
11. Wang, R. F.; Cao, W. W.; Cerniglia, C. E. PCR detection and quantitation of predominant anaerobic bacteria in human and animal fecal samples. *Appl Environ Microbiol* **1996,** *62*, 1242-1247.
12. Rinttila, T.; Kassinen, A.; Malinen, E.; Krogius, L.; Palva, A. Development of an extensive set of 16S rDNA-targeted primers for quantification of pathogenic and indigenous bacteria in faecal samples by real-time PCR. *J Appl Microbiol* **2004,** *97*, 1166-1177.
13. Larsen, N.; Vogensen, F. K.; van den Berg, F. W.; Nielsen, D. S.; Andreasen, A. S.; Pedersen, B. K.; Al-Soud, W. A.; Sorensen, S. J.; Hansen, L. H.; Jakobsen, M. Gut microbiota in human adults with type 2 diabetes differs from non-diabetic adults. *PloS One* **2010,** *5*, e9085.

14. Collado, M. C.; Delgado, S.; Maldonado, A.; Rodriguez, J. M., Assessment of the bacterial diversity of breast milk of healthy women by quantitative real-time PCR. *Lett Appl Microbiol* **2009,** *48*, 523-528.
